# Effect of Repeated Autoclave on Hardness and Tensile Strength of Polypropylene/Natural Rubber Developed for Rubber Dam Clamp

**DOI:** 10.3390/polym17020143

**Published:** 2025-01-08

**Authors:** Thitaporn Nonthiphalang, Panupat Phumpatrakom, Paphavarin Rangsantham, Panjaporn Wongwitthayakool, Chakrit Sirisinha, Nantawan Krajangta

**Affiliations:** 1 Division of Restorative Dentistry, Faculty of Dentistry, Thammasat University, Pathumthani 12121, Thailand; thitaporn.non@dome.tu.ac.th; 2 Division of Endodontics, Faculty of Dentistry, Thammasat University, Pathumthani 12121, Thailand; panupatp@tu.ac.th (P.P.); paphavarin.ran@dome.tu.ac.th (P.R.); 3 Division of Oral Biology, Faculty of Dentistry, Thammasat University, Pathumthani 12121, Thailand; panja_w@tu.ac.th; 4 Rubber Technology Research Center (RTEC), Faculty of Science, Mahidol University, Nakhon Pathom 73170, Thailand; chakrit.sir@mahidol.ac.th

**Keywords:** polypropylene, natural rubber, hardness, tensile strength, sterilization, rubber dam clamp

## Abstract

In this study, we aimed to explore the feasibility of utilizing polypropylene (PP) and natural rubber (NR) blends as alternative materials for rubber dam clamps in dentistry. The hardness of various PP/NR blend ratios was compared to the commercial product SoftClamp^TM^. Selected blend ratios exhibiting hardness values resembling rigid plastic were further evaluated for hardness and tensile strength after undergoing 1, 5, and 10 autoclave cycles. One-way ANOVA test results found that the PP/NR blends exhibited significantly lower hardness (*p* < 0.001) than the commercial SoftClamp^TM^. PP/NR ratios of 100/0, 90/10, and 80/20 exhibited hardness levels equivalent to 82%, 80%, and 75% of SoftClamp^TM^, respectively. Two-way ANOVA revealed significant reductions in hardness (*p* < 0.001) and tensile strength (*p* < 0.001) with increasing NR content across all autoclave cycles. However, post-autoclave assessments at 1, 5, and 10 cycles demonstrated no statistically significant differences in tensile strength (*p* = 0.058) and hardness (*p* = 0.52) for PP/NR ratios of 100/0, 90/10, and 80/20 compared to their pre-autoclave states. The NR content within the PP/NR blends decreased hardness and tensile strength, while autoclaving did not significantly affect the hardness and tensile strength of the PP/NR blends.

## 1. Introduction

Rubber dam clamps have been used in dentistry for a long time, with their origin dating back to 1864 when they were first conceived by Barnum [1]. Over time, they have become an essential standard of care in isolating specific teeth and maintaining a contamination-free working field during restorative and endodontic treatments. With the COVID-19 pandemic, the importance of rubber dams and clamps has been further emphasized, highlighting their crucial role in proper clinical practice [2]. Despite their established benefits, traditional stainless-steel clamps have some drawbacks, such as trauma to gingival tissues, clamp slippage, poor peripheral sealing in severely worn teeth, corrosion, and limitations in bending resistance after multiple uses [3,4]. To address these challenges, flexible (non-metallic) clamps like the SoftClamp^TM^ by Kerr Corp., Orange, CA, USA, have been proposed to reduce patient discomfort and iatrogenic damage [5]. However, the prohibitive cost of non-metallic clamps limits their widespread use.

This study focuses on exploring a new alternative for developing reusable non-metallic clamps using polypropylene (PP) and natural rubber (NR) blends. PP is known for its biocompatibility and advantageous properties when used in numerous biomedical products, high heat distortion temperature, autoclavable features, and financial advantages. NR can be used to toughen PP while maintaining the stiffness and processability of PP [6,7]. The combination of PP and NR results in thermoplastic elastomers (TPEs), combining characteristic properties of both thermoplastics and elastomers [8]. This approach offers potential benefits to both the economy of rubber-producing regions and local farmers. While PP and NR have been reported in various medical fields [9,10], their specific application in dental rubber dam clamps has been scarcely explored, with only one study to date [11]. This research aims to address this gap by investigating the properties of PP/NR blends for dental applications, thereby highlighting the novelty and innovation of our work in the dental field.

Autoclaving sterilization is typically used to sterilize medical devices by creating saturated steam under pressure to kill microorganisms [12]. It is considered the most robust and cost-effective method for sterilizing medical devices [13]. However, autoclaving sterilization can affect TPEs’ properties due to the heat, pressure, and moisture that the materials are subjected to during the sterilization process [14].

Chemical and thermal degradation processes can occur in TPEs when they are exposed to high temperatures and pressures during autoclaving. This degradation may involve chain scission, crosslinking, and oxidation, leading to changes in the hardness or durometer of the TPEs [15]. Moreover, autoclaving can induce microstructural changes in TPEs, including changes in crystallinity, morphology, and phase separation [16].

The Durometer or Shore durometer, specifically Shore D hardness, is typically used as a standard for measuring the hardness of non-metallic materials such as polymer, rubber, and elastomer [17]. Shore hardness is also a measure of the resistance of a material to penetration [18]. This test is valuable for assessing the material’s capacity to withstand deformation, particularly during clinical use, where rubber dam clamps are exposed to significant forces and pressures from rubber dam forceps. On the other hand, tensile testing evaluates the material’s ability to resist forces applied along its length, providing insights into its strength and elasticity [19]. This test helps determine the maximum load a material can withstand before breaking, which is crucial for assessing the structural integrity and safety of rubber dam clamps that do not fracture and remain securely in place during dental procedures, especially when clamped onto teeth.

In this study, the PP/NR blend was prepared at various weight ratios of 100/0, 90/10, 80/20, 70/30, 60/40, and 50/50. The hardness of the blends was measured and compared with a commercial non-metallic rubber dam clamp (SoftClamp^TM^). The blend ratios exhibiting hardness values in the scale of rigid plastic were selected to measure the hardness and tensile strength after the autoclaving sterilization. The influence of the autoclave cycles on the hardness and tensile strength of PP/NR blends was investigated to assess applicability in clinical settings. The hypotheses considered are as follows: (i) the increasing NR concentrations would affect the hardness and tensile strength of the PP/NR blends; (ii) the hardness and tensile strength of the PP/NR blends would be changed by increasing the autoclaving cycles.

## 2. Materials and Methods

### 2.1. Experimental Design

In this study, the hardness and tensile strength of PP/NR at various blend ratios (100/0, 90/10, 80/20, 70/30, 60/40, and 50/50) were assessed. The hardness was tested in comparison with a commercial non-metallic rubber dam clamp (SoftClamp^TM^, Kerr Corp., Orange, CA, USA). The blend ratios exhibiting excessive softness and immeasurability by Shore D hardness were excluded. The PP/NR blend ratios demonstrating hardness values in the level of rigid plastic were selected for investigating the effects of autoclave cycles (i.e., 1, 5, and 10 autoclave cycles).

**Figure 1:**
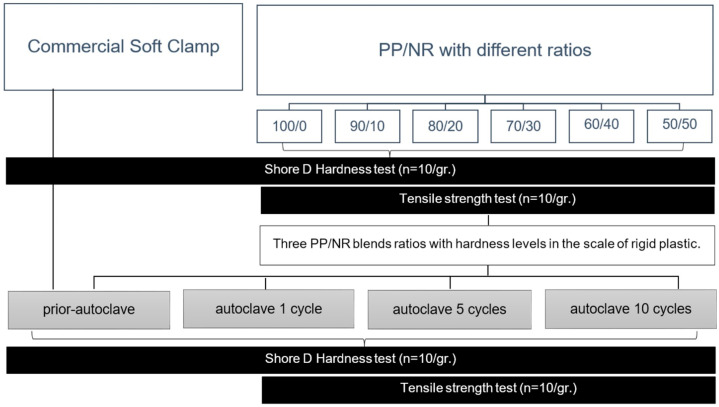
Experimental design.

The investigation involved an examination of the ASTM D2240 and ASTM D638 standards for Shore D hardness and tensile testing, respectively [20,21]. According to the ASTM D638 standard, a minimum of 10 specimens per group was recommended for the tensile test [21]. Consequently, the sample size for each experimental group in this study was established at 10 specimens. The study design, outlined in Figure 1, provides a comprehensive summary of the experimental framework. Additionally, the morphological characteristics of randomly selected tensile specimens of the PP/NR blend before and after 10 autoclave cycles were analyzed using a scanning electron microscope (SEM).

### 2.2. PP/NR Blended Preparation

In this study, polypropylene (EL-Pro, SCG Performance Chemical, Bangkok, Thailand) with a melting temperature of 169 °C was used as the major component. Natural rubber (STR5L) supplied by Kij Paiboon Chemical Ltd. was used as a toughening agent. The PP/NR blends were prepared at ratios of 100/0, 90/10, 80/20, 70/30, 60/40, and 50/50 by weight using the melt mixing technique in a laboratory-size internal mixer (Haake Rheocord 90 Fisons, Berlin, Germany) at 175 °C. The PP was first charged to the mixer and allowed to melt for 3 min, followed by the addition of NR. Mixing continued for 7 min before discharging.

The blends were shaped to the desired dimensions for hardness and tensile tests using a hydraulic hot press (Chareon Tut, Samut Prakan, Thailand) at 175 °C for 8 min and cooled at room temperature under pressure.

### 2.3. Hardness Test

The hardness of the test specimens with dimensions of 48.0 × 24.0 × 6.0 mm^3^ was assessed following ASTM D2240 using a Shore D durometer (Bareiss Prüfgerätebau GmbH, Oberdischingen, Germany). Before testing, the test specimens were conditioned at room temperature (25 °C) for 24 h. During the test, the indenter was pressed perpendicularly into the surface of the specimen using a test load of 4536 g. The hardness reading was recorded after the indenter made firm contact with the specimen surface within 1 s to ensure accuracy. Five hardness measurements were taken for each specimen at different positions, with a minimum separation of 6.0 mm and a distance of at least 12.0 mm from any edges, as per ASTM D2240.

### 2.4. Tensile Testing

**Figure 2:**
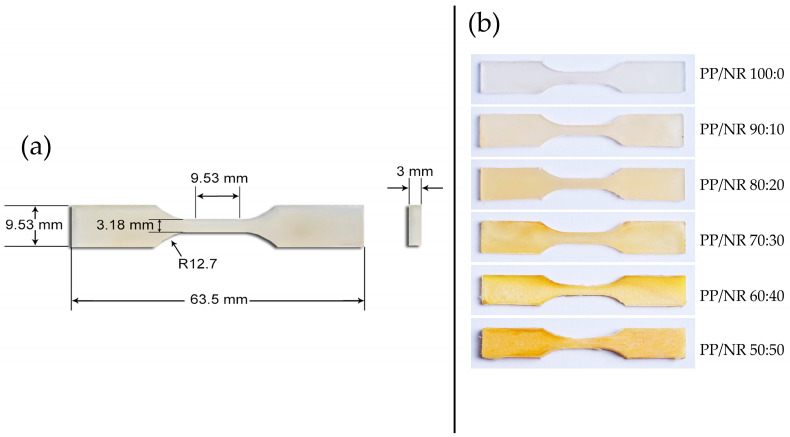
Tensile strength specimen. (**a**) Specimen dimensions; (**b**) the different blend ratios of the specimens.

The tensile test specimens were prepared according to the ASTM D638 specimen dimensions (Figure 2). The dimensions of the specimen were measured by a caliper (Mitutoyo, Kawasaki, Japan) with an accuracy of at least 0.01 mm. The tensile test was conducted using a Shimadzu universal testing machine (Shimadzu Corp., Kyoto, Japan) following ASTM D638 (Type V specimen dimensions) with a crosshead speed of 1 mm/min. The sample was deformed until fracture, and the force at the fracture was calculated to determine the tensile strength.

### 2.5. Autoclave Steam Sterilization

This study explored the impact of autoclave cycles on the hardness and tensile strength of three specifically selected ratios of PP/NR, chosen for their hardness values that closely resembled those of a commercially available SoftClamp^TM^ to investigate the effect of multiple autoclave cycles (pre-autoclave and after 1, 5, and 10 cycles in the autoclave).

Specimens of each PP/NR ratio were subjected to steam sterilization using an autoclave (MML-Bester, Med tech Ltd., Bangkok, Thailand) at 121 °C (250 °F) for 20 min under a pressure of 0.103 MPa (15 psi) and a nearly 100% humidity level. Ten specimens of each blend ratio were subjected to 1, 5, or 10 autoclave cycles. The specimens were then left to dry overnight before the next autoclave cycle.

### 2.6. Morphological Analysis (SEM)

A tensile specimen from each of the three specifically selected ratios of PP/NR, corresponding to pre-autoclave and after 10 autoclave cycles, was randomly chosen for morphological analysis. The topography of the fracture surface of the tensile test specimen was sputter-coated with gold in a vacuum chamber (JEOL Smart Coater, JEOL Ltd., Tokyo, Japan) before observation. The surface morphology was obtained using a scanning electron microscope (SEM) (JEOL JCM-6000, JEOL Ltd., Tokyo, Japan) at ×500 and ×2000 magnification. The SEM was operated at an accelerating voltage of 10 kV.

### 2.7. Statistical Analysis

The results of all groups were analyzed using SPSS 26 software for Windows (SPSS Inc., Chicago, IL, USA), setting the confidence level at 95% and significance level at α = 0.05. For hardness and tensile data, the normality of data distribution was confirmed using the Shapiro–Wilk test, and variance homogeneity of all groups was confirmed with Levene’s test, except for the tensile data of each PP/NR ratio after 1, 5, and 10 cycles, where homogeneity of variance was not validated. One-way ANOVA was employed to examine the effects of different materials (PP/NR at various ratios and SoftClamp™). Furthermore, a two-way ANOVA was conducted to assess the influence of different materials (PP/NR at ratios of 100/0, 90/10, and 80/20) and distinct autoclave cycles (pre-autoclave and 1, 5, and 10 cycles). Multiple comparisons for hardness were performed before and after autoclaving, and tensile data before autoclaving were obtained using Tukey’s post hoc test. Dunnett’s T3 was also considered for analyzing tensile data after autoclaving.

## 3. Results

### 3.1. Hardness Test

All specimens, comprising various ratios of PP/NR, were sufficiently rigid to be measured with the Shore D hardness, and the statistical test for mean and standard deviation results using both one-way and two-way ANOVA are presented in Table 1.

### 3.2. Tensile Test 

The tensile test of the specimens was conducted according to ASTM D638 standards. The statistical test for mean and the standard deviation results of tensile strength using both one-way and two-way ANOVA are presented in Table 2.

### 3.3. Surface Morphology

**Figure 3:**
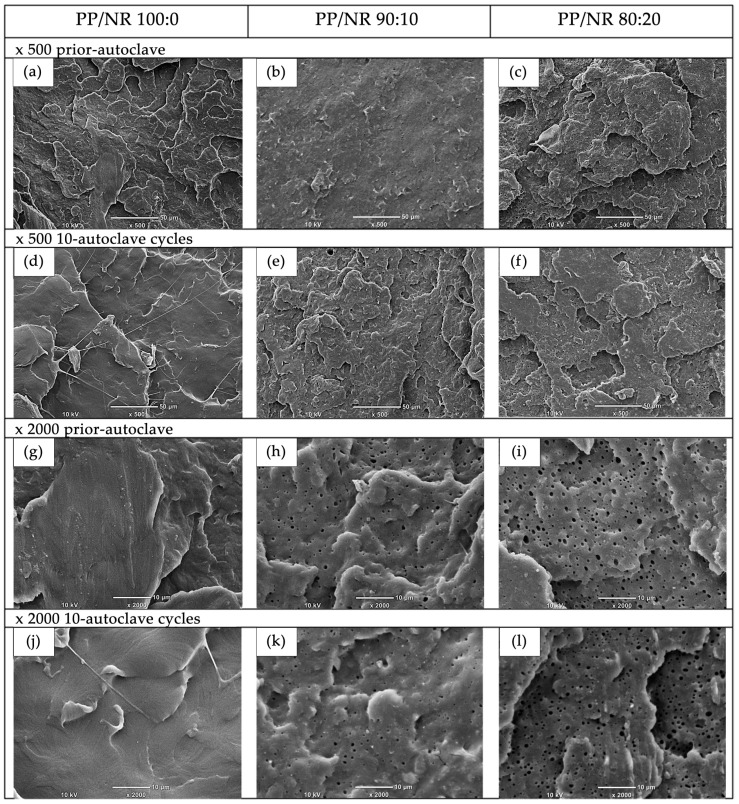
SEM images of the cross-sectional surface of tensile specimens before and after 10 autoclave cycles. SEM image at ×500 magnification of pre-autoclave specimens (**a**–**c**) and specimens after 10 autoclave cycles (**d**–**f**). SEM image at ×2000 magnification pre-autoclave specimens (**g**–**i**) and specimens after 10 autoclave cycles (**j**–**l**). Subfigures represent PP/NR blend ratios (100:0, 90:10, and 80:20).

The scanning electron microscope (SEM) images of the cross-sectional surface of the tensile test specimens are shown in Figure 3. The distinct morphological characteristics between the pure PP and PP/NR blends were observed. Most of the surfaces reveal spherical-shaped voids, attributed to the extraction of polypropylene (PP) or natural rubber (NR) domains. Notably, the pure PP fractography deviates from this pattern.

## 4. Discussion

The obtained results support the first hypothesis that the increase in NR content significantly affects the hardness and tensile strength of PP/NR blends. However, the findings do not agree with the second hypothesis, proposing the variations in hardness and tensile strength of the PP/NR blends with increasing autoclave cycles.

Rubber dam clamps help isolate the tooth or teeth being worked on, ensuring the area remains dry and saliva-free during dental treatment. Therefore, the hardness of rubber dam clamps should be balanced to provide optimal performance during dental procedures while ensuring patient comfort and safety. The traditional metal dental clamps have served dentists well for decades; they are often made from 420 carbon martensitic steel, with Rockwell C hardness values ranging from 49 to 56 [18,19]. Additionally, numerous studies have shown the adverse effects associated with metal clamps, potentially linked to the rigidity of the metal, such as pain, discomfort, and enamel loss [22]. As mentioned earlier, commercial non-metallic clamps have been utilized to reduce the limitations of metal dental clamps.

In this study, a commercial rubber dam clamp, SoftClamp^TM^, made from polyetheretherketone (PEEK) [22] was selected as the reference standard data due to its widespread use and recognition as a leading brand in the non-metallic category within the dental clamp market. Furthermore, due to the absence of established criteria or universally accepted standards defining the optimal hardness level for non-metallic rubber dam clamps suitable for clinical dental procedures, this choice was deemed appropriate. However, it is important to note a limitation regarding the specimen preparation. Because of the high melting temperature of the PEEK in the commercial soft clamp, a remelting of the commercial soft clamp for shaping a tensile test specimen is not possible. Therefore, only hardness could be measured, and it served as a reference in this study.

**Figure 4:**
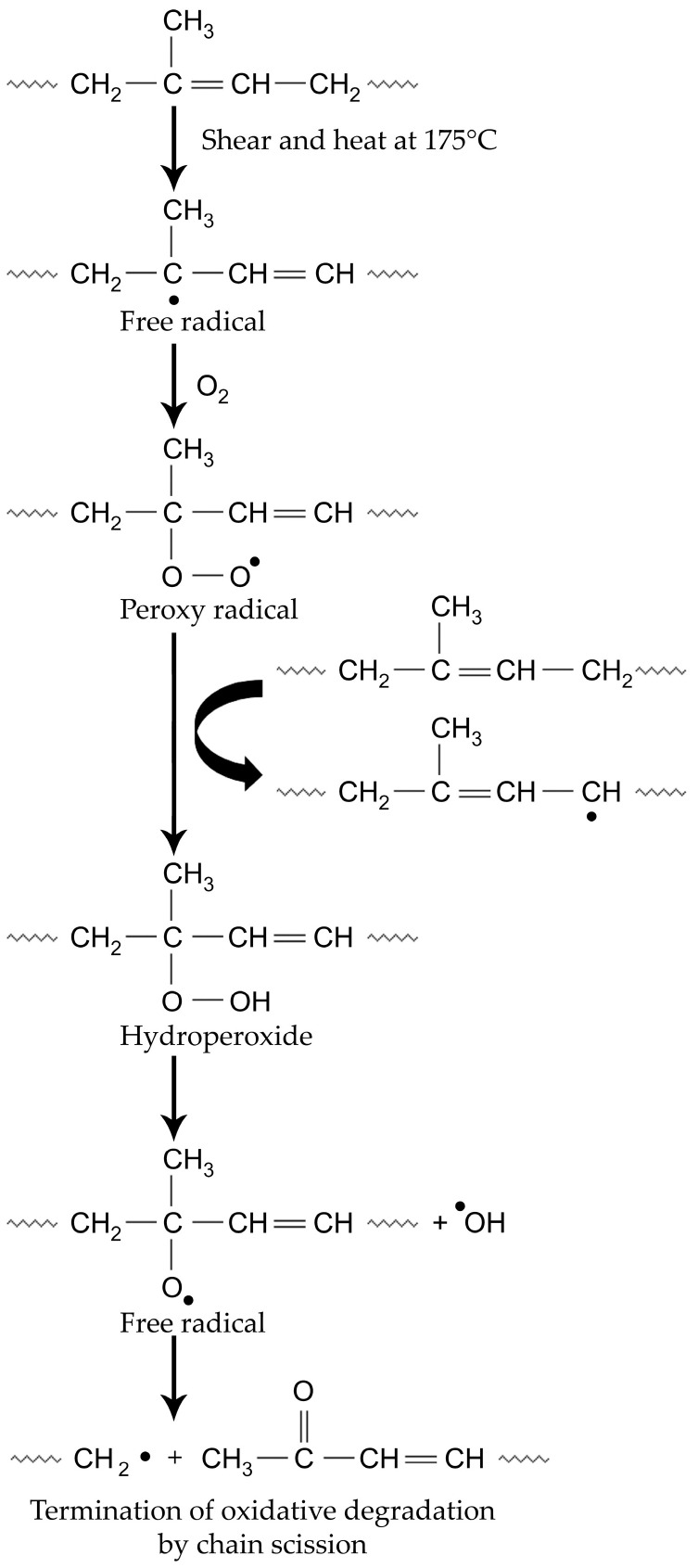
The reaction scheme of thermo-oxidative degradation of the NR phase during blending at a high temperature of 175 °C.

From Table 1, we can see that the average hardness of the SoftClamp^TM^ was 85.70 ± 0.42 Shore D, which agrees well with that of PEEK [23]. Expectedly, the SoftClamp^TM^ demonstrated a statistically significant higher hardness compared to both pure PP and PP/NR blends at all tested ratios (*p* < 0.001). The incorporation of NR into PP led to a statistical reduction in both hardness and tensile strength (*p* < 0.001). Thermo-oxidative degradation of the NR phase during blending at a high temperature of 175 °C can be used as the explanation. The presence of high numbers of double bonds on the NR backbone gives rise to a susceptibility to oxidation, which causes main chain scission, resulting in a deterioration of the mechanical properties, as seen in Figure 4 [24,25].

The higher the NR loading, the higher the degraded composition, leading to lower hardness. The PP/NR blend ratios of 100/0, 90/10, and 80/20 displayed Shore D hardness levels of 70.4 ± 0.50, 68.45 ± 0.29, and 63.95 ± 0.34, respectively, which are considered to be remain similar to the conventional hardness of PP [26] and are at the level of rigid plastic [27]. Consequently, these three blends were selected for hardness and tensile assessment post-autoclave sterilization. Eskibağlar et al. found that stainless-steel clamps caused much higher stress on the molar than SoftClamps™, with an 85% difference in stress levels [22]. However, the study reported by Pottammal et al. showed no statistically significant difference in pain response between children subjected to commercial soft clamps and metal clamps [28]. Therefore, utilizing PP/NR blends with hardness levels slightly lower than that of the commercial soft clamp, on the scale of rigid plastic, for clamp production could potentially reduce stress distribution on the tooth and reduce patient discomfort. However, further research is necessary to validate this assertion.

In a recent study in 2021, Fischer and Howells investigated the reusability of autoclaved 3D-printed polypropylene (PP) compared to glass-filled polypropylene composite (GFPP) [16]. Their research indicated that the higher autoclave temperature of 134 °C was excessive, resulting in the melting of PP and GFPP within merely 2–3 autoclaving cycles. Conversely, both PP and GFPP cubes displayed minor alterations, amounting to less than 1%, in mass and volume following one, four, seven, and ten autoclaving cycles at 121 °C [16]. Therefore, the present study focused on evaluating the properties of PP/NR post-autoclaving, opting for a temperature of 121 °C for experimentation. The results reveal that, after autoclaving for 1, 5, and 10 cycles, there were no significant changes in the shape, hardness, and tensile strength of pure PP and PP/NR blends at the blend ratios of 90/10 and 80/20. Interestingly, the increase in the autoclaving cycle did not significantly impact the hardness and tensile strength of the blends. Although statistical analysis indicates no significant changes in hardness and tensile strength after autoclaving, the test results show an increase in tensile strength after the first autoclave cycle (from 36.78 to 46.85%) followed by a subsequent decrease (not statistically significant) in values for the pure PP. The initial increase can be attributed to the annealing effect and potential crosslinking within the PP matrix, temporarily enhancing tensile strength. However, subsequent cycles led to thermo-oxidative degradation and microstructural changes, resulting in a decrease in tensile strength, similar to the PP/NR blends at blend ratios of 90/10 and 80/20.

SEM images of the fractured surfaces of the various PP/NR blends are shown in Figure 3. The NR phase is dispersed in the PP matrix as small droplets, and the NR phase slightly increases, both in terms of size and quantity, with increasing NR content. After autoclaving, the blend morphology did not significantly change, which agrees well with the hardness and tensile test results, as discussed previously. In other words, autoclaving has no adverse effect on the tensile strength of PP/NR blends. Similar findings were also reported in [29,30]. Giebink et al. (1996) [30] demonstrated that 30 sterilization cycles did not affect the stiffness or dimensions of rubber dam clamps. By contrast, Chhabra et al. (2018) [29] reported increased tensile strength in metal rubber dam clamps that had undergone three hundred cycles of use and sterilization. Therefore, PP/NR blends with ratios of 90/10 and 80/20 may serve as viable alternatives for producing non-metal clamps, although further investigation is warranted.

This study has limitations due to the absence of established standards for hardness and tensile strength values specific to non-metallic dental dam clamps. As a result, the investigation still relied on comparative analyses with commercial products as a reference standard. Additionally, autoclaving was limited to 10 cycles in this study, and future research should assess PP/NR blend properties after autoclaving with higher cycles. Moreover, it is essential to prepare specimens resembling dental clamps and conduct relevant clinical tests, such as attachment to teeth, to simulate clinical usage and assess force distribution on the clamp and teeth. Detailed studies on the microstructural changes due to autoclaving, using advanced imaging techniques such as transmission electron microscopy (TEM) and atomic force microscopy (AFM), are also crucial. These techniques can provide deeper insights into the morphological and structural changes at the nanoscale, which are essential for understanding the long-term performance and durability of PP/NR blends in clinical applications. Additionally, rubber dam clamps do not directly make contact with soft tissue in the oral cavity; they only do so with teeth during procedures and are removed afterwards. PP and natural rubber have been used in many medical fields [9,10], and their biocompatibility is well documented. However, there are reports of allergies to natural rubber [10], so it is advisable to avoid using these materials in patients with known allergies.

## 5. Conclusions

A lower proportion of NR content within PP/NR blends results in significantly higher hardness and tensile strength, before and after autoclaving for 1, 5, and 10 cycles. Notably, the autoclaving process does not affect the hardness and tensile strength changes of pure PP and PP/NR blends at ratios of 90/10 and 80/20.

## Figures and Tables

**Table 1:** The Shore D hardness results using one-way and two-way ANOVA.

Material	Mean (SD) of Hardness (Shore D)				*p*-Value of Two-Way ANOVA
	Number of Autoclave Cycles				
	Prior	1 Cycle	5 Cycles	10 Cycles	
PP/NR 100:0	70.4 (0.50) aA	70.0 (0.25) aA	70.0 (0.47) aA	69.75 (0.28) aA	Diff among PP/NR ratios < 0.001 Diff among cycles = 0.52
PP/NR 90:10	68.45 (0.29) bA	65.85 (0.25) bA	66.60 (0.27) bA	67.20 (0.25) bA	
PP/NR 80:20	63.95 (0.34) cA	64.30 (0.34) cA	63.20 (0.36) cA	63.80 (0.23) cA	
PP/NR 70:30	57.10 (0.48) d	N/A			
PP/NR 60:40	54.00 (0.30) e				
PP/NR 50:50	45.00 (0.21) f				
SoftClamp^TM^	85.70 (0.42) g				
*p*-value of one-way ANOVA	<0.001				

Identical lowercase letters within a column indicate no statistically significant difference between the materials (*p* > 0.05). Identical uppercase letters within a row indicate no statistically significant difference between the autoclave cycles (*p* > 0.05). N/A: not applicable (not all the specimens were subjected to testing).

**Table 2:** The tensile strength results using One-way and Two-way ANOVA.

Material	Mean (SD) of Tensile Strength (MPa)				*p*-Value of Two-Way ANOVA
	Number of Autoclave Cycles				
	Prior	1 Cycle	5 Cycles	10 Cycles	
PP/NR 100:0	36.78 (0.52) aA	46.85 (0.84) aA	35.29 (0.23) aA	35.72 (0.31) aA	Diff among PP/NR ratios < 0.001 Diff among cycles = 0.058
PP/NR 90:10	29.79 (0.62) bA	29.34 (0.62) bA	27.62 (0.18) bA	28.79 (0.23) bA	
PP/NR 80:20	26.13 (0.38) cA	24.28 (0.37) cA	24.75 (0.39) cA	25.09 (0.22) cA	
PP/NR 70:30	20.69 (0.33) d	N/A			
PP/NR 60:40	19.45 (0.43) d				
PP/NR 50:50	15.00 (0.37) e				
*p*-value of one-way ANOVA	<0.001				

Identical lowercase letters within a column indicate no statistically significant difference between the materials (*p* > 0.05). Identical uppercase letters within a row indicate no statistically significant difference between the autoclave cycles (*p* > 0.05). N/A: not applicable (not all the specimens were subjected to testing).

## Data Availability

Data are contained within the article.
